# Optimal Distributed Finite-Time Fusion Method for Multi-Sensor Networks under Dynamic Communication Weight

**DOI:** 10.3390/s23177397

**Published:** 2023-08-24

**Authors:** Hang Yu, Keren Dai, Qingyu Li, Haojie Li, He Zhang

**Affiliations:** 1 School of Mechanical Engineering, Nanjing University of Science and Technology, Nanjing 210094, China; hangyu@njust.edu.cn (H.Y.); hezhangz@njust.edu.cn (H.Z.); 2 North Information Control Research Academy Group Co., Ltd., Nanjing 211153, China; liqy14@tsinghua.org.cn

**Keywords:** distributed Kalman filter, sensor networks, dynamic communication weight, finite-time consensus

## Abstract

Aiming at the problem of distributed state estimation in sensor networks, a novel optimal distributed finite-time fusion filtering method based on dynamic communication weights has been developed. To tackle the fusion errors caused by incomplete node information in distributed sensor networks, the concept of limited iterations of global information aggregation was introduced, namely, fast finite-time convergence techniques. Firstly, a local filtering algorithm architecture was constructed to achieve fusion error convergence within a limited number of iterations. The maximum number of iterations was derived to be the diameter of the communication topology graph in the sensor network. Based on this, the matrix weight fusion was used to combine the local filtering results, thereby achieving optimal estimation in terms of minimum variance. Next, by introducing the generalized information quality (GIQ) calculation method and associating it with the local fusion result bias, the relative communication weights were obtained and embedded in the fusion algorithm. Finally, the effectiveness and feasibility of the proposed algorithm were validated through numerical simulations and experimental tests.

## 1. Introduction

### 1.1. Motivation and Related Work

With the development of sensor technology, sensor networks have been widely used in various fields such as environmental monitoring [1], target tracking [2], and autonomous driving [3]. The important content of sensor networks research is the state estimation of filter output. Common information fusion algorithms applied to state estimation in multi-sensor networks including weighted averaging [4,5], multi-Bayesian estimation methods [6,7], Kalman filtering (KF) [8,9].

There are currently two typical fusion structures for the KF algorithm [10]. The first one is the centralized Kalman filtering (CKF) fusion method, where all information is gathered at a central node for fusion. This method minimizes information loss and achieves high fusion accuracy. However, the shortcomings of this approach are also obvious, because it needs to process a large amount of information, which brings a heavy computational burden to the central node. In addition, the centralized fusion method has poor robustness. If other sensor nodes fail and provide significantly biased measurement information, the final fusion result will be seriously affected. The second one is the distributed Kalman filtering (DKF) fusion method, which differs from the centralized fusion method in that it doesn’t rely on a central node. In the distributed fusion method, each sensor node performs local preprocessing of its own measurement information, and then performs fusion. Therefore, in the absence of data faults, the fusion accuracy of distributed fusion methods is usually lower than that of centralized fusion, but its strong robustness makes fault detection and isolation easier.

It is expected to obtain a stable fusion state quantity in the shortest possible time for target tracking and environmental monitoring. Therefore, information fusion algorithms are required to have consistent control capabilities for fusion errors. In [11,12,13,14], there is a common feature in these studies: the average consensus strategy is used, that is, theoretically, large numbers of iterations are required to achieve asymptotic convergence, but for most sensor network application scenarios, there is a high demand for real-time performance of fusion results. Therefore, it requires that the estimation error of the filtering algorithm should have the characteristics of finite-time convergence. To improve these shortcomings, the DKF algorithm with finite-time convergence is proposed in [15,16,17,18]. Using the maximum consensus technique, a finite-time KF algorithm with local unobservability was proposed in [15]. In [16,17], using maximum consistency technology, the sensor network can achieve consensus in finite time. This paper adopts a fusion processing method that achieves finite-time convergence of estimation errors to enhance the practicality of the algorithm. In [18], a finite-time DKF algorithm was proposed, which can achieve dynamic monitoring of linear discrete dynamic systems using a sensor network with active sensors and some idle sensors.

Only using the DKF algorithm does not easily meet the authenticity in various application scenarios such as environmental monitoring, target tracking and intelligent transportation. Many scholars have increased the weight of the fusion process to enhance the authenticity of sensor networks in various applications. In [19,20,21,22,23], fusion weights have been incorporated into information fusion algorithms, but the approaches to handle these weights vary. A global KF and long short-term memory (LSTM)-based measurement variance data fusion method were designed in [19] by incorporating an adaptive truncation mechanism to determine optimal weights. In [20,21], to address the impact of significant variations in the measured data on fusion accuracy, the weight coefficients are dynamically adjusted in real-time to achieve an improvement in the accuracy of prediction values. In order to achieve a good fusion effect and minimize filtering errors, an algorithm using both the KF and an unbiased finite impulse response filter are proposed in [22]. The switching between the two filters is accomplished by applying probabilities to match the weights. In [23], a set of constrained optimization problems is solved using mathematical induction, set theory, and convex optimization methods to obtain the fusion weights for the filters. However, the aforementioned reference did not consider the communication weights of the sensor network, which has certain ideal constraints.

### 1.2. Comparison and Main Contributions

Through the literature review mentioned above, a comparison of the main advantages and disadvantages of several fusion algorithms mentioned in this paper is presented. As shown in Table 1:

Based on the above analysis, in this work, a distributed structure is used to design a fusion algorithm, which enhances the robustness of the algorithm. Based on this, a finitetime control technique is introduced to design a distributed optimal KF fusion algorithm, which achieves fast convergence of fusion error and optimal estimation with minimum variance. Furthermore, the dynamic communication weights associated with the local fusion result bias are designed based on GIQ computing technology. This enhances the feasibility of the fusion algorithm in practical scenarios.

Specifically, the main contributions of this paper are as follows:

- First, in contrast to references [12,13,14,15,18,26,27,28,29], which only consider fusion accuracy or the consistency of the fusion process without achieving rapid convergence of fusion errors and optimal estimation, this paper introduces fast finite-time convergence techniques and matrix weight fusion techniques. It achieves finite-time convergence of fusion errors (the maximum convergence iterations being the graph diameter) and optimal estimation in terms of minimum variance.
- Second, unlike [19,20,21,22,23,30] that are just concerned with the weight fusion of filtering results in the key step about the algorithm and do not focus on the communication weight settings in the fusion process or the dynamic correlation between the implementation weight and filtering results. In response to this, a dynamic communication weight generation technique called GIQ calculation is introduced to calculate bias of the current local fusion result in real-time. This technique is used to represent the local filtering effect.
- Last but not least, unlike most studies which only validate the proposed algorithm through simulations, this paper verifies the feasibility and execution accuracy of the proposed optimal distributed finite-time convergence fusion algorithm through both numerical simulations and experiments.

The remainder of this paper is organized as follows. Section 2 describes necessary preliminaries, notations. The multi-source information fusion algorithm with finite-time convergence, optimality and convergence are presented in Section 3. Simulation analysis and experiments of the fusion algorithm are presented in Section 4. Finally, conclusions are given in Section 5.

## 2. Preliminaries and Notations

### 2.1. Problem Statement

This paper studies the problem of precise observation of target states in distributed sensor networks. The state of the observed target is modeled as the following discrete linear system:

(1) $$x_{k+1}=\Phi_{k}x_{k}+w_{k},$$

where $x_{k}\in R^{n_{x}}$ is the target state to be estimated, *k* denoted as instant, $\Phi_{k}\in R^{n_{x}\times n_{x}}$ is a known system matrix, and $w_{k}\in R^{n_{x}}$ is the process noise, which is assumed to be Gaussian white noise with zero-mean. According to fusion theory, a fusion system fuses estimated values with observed values, therefore there exists an observation equation that can be expressed as:

(2) $$y_{k}^{i}=H_{k}^{i}x_{k}+v_{k}^{i},i=1,2,\ldots ,n,$$

where $y_{k}^{i}\in R^{m_{i}}$ is the measurement value of sensor *i*, $H_{k}^{i}\in R^{m_{i}\times n_{x}}$ is a known measurement matrix, and $v_{k}^{i}\in R^{m_{i}}$ is the measurement noise, which is also assumed to be Gaussian white noise with zero-mean, *n* represents the number of sensors.

For the Gaussian white noise in (1) and (2) and the initial state $x_{0}$ of the sensors, the following assumptions are made:

**Assumption A1.**  *The process noise $w_{k}$ and measurement noise $v_{k}^{i}$ are independent, uncorrelated, and have no autocorrelation. The covariance matrices are denoted as $Q_{k}$ and $R_{k}^{i}$, respectively. Then:*

(3) $$E[v_{k}^{i}{\left(v_{t}^{j}\right)}^{T}]=0,\;i\neq j;\forall k,t,$$

(4) $$E\left\{\left[\begin{array}{c} w_{k}\\ v_{k}^{i} \end{array}\right]\left[\begin{array}{cc} w_{k}^{T} & {\left(v_{k}^{i}\right)}^{T} \end{array}\right]\right\}=\left[\begin{array}{cc} Q_{k} & 0\\ 0 & R_{k}^{i} \end{array}\right]\delta_{kt},$$

*where E(x) is represented as the expectation operation on x. In the above equation, it calculates the expectation of the expression inside the parentheses. The superscript T indicates the operation of transpose and j denoted as the sensor node. $\delta_{tk}$ is the Kronecker function, which has the following form:*

(5) $$\delta_{tk}=\left\{\begin{array}{cc} 1, & \mathrm{if}\;k=t\\ 0, & \mathrm{if}\;k\neq t. \end{array}\right.$$

**Assumption A2.**  *The initial state $x_{0}$ of the sensor is uncorrelated with noise $w_{k}$ and $v_{k}^{i}$, then:*

(6) $$\begin{array}{c} E\left[x_{0}\right]=\mu_{0}\\ E\left[\left(x_{0}-\mu_{0}\right){\left(x_{0}-\mu_{0}\right)}^{T}\right]=P_{0}, \end{array}$$

*where $\mu_{0}$ and $P_{0}$ are the expectation and covariance of initial state $x_{0}$, respectively.*

### 2.2. Communication Topology

As this subsection assumes a scenario of multi-sensor information fusion, there are multi-sensors serving as input sources for detection information. And all sensor nodes possess routing capabilities. To obtain the most accurate global information under different scenarios, a communication topology network is established between sensor nodes to transmit the detected information from one sensor to another. Therefore, the communication topology among sensors is described as G=(V,E,A), which consists of a node set V={1,2,…,n}, an edge set E⊆V×V, and an adjacency matrix $A=\left[a_{ij}\right]\in R^{n\times n}$, i,j∈V. For an undirected graph, that is, (i,j)∈E⇔(j,i)∈E, this means that node *i* and node *j* can perceive each other. And the elements of the adjacency matrix A satisfy $a_{ij}=a_{ji}>0$, otherwise, $a_{ij}=0$. In this case, node *j* is called a neighbor of node *i* [26,31,32].

**Figure 1:**
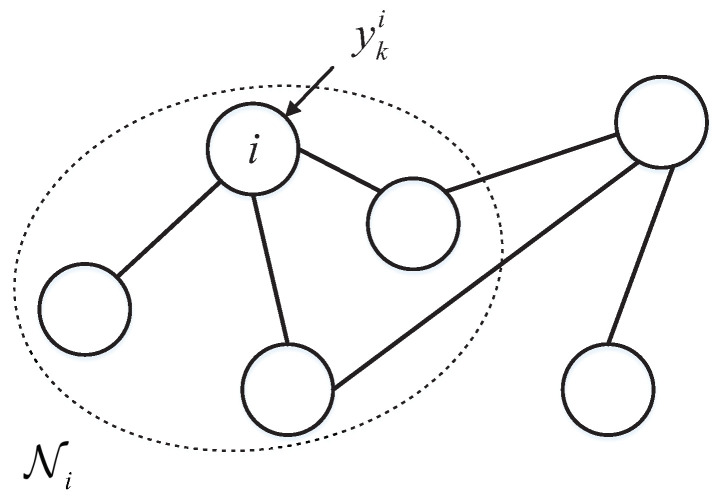
Schematic of neighbor node set of sensor node *i*.

In particular, it is allowed to have individual sensors for independent detection, that is, $a_{ii}=1$. The dynamic weight matrix at instant *k* is defined as $W=[\omega_{ij}\left(k\right)]\in R$ to represent the information exchange weights between node *i* and node *j*. All inneighbors of node *i* is denoted as $N_{i}=\left\{j\in V|\left(j,i\right)\in E\right\}$, it can be represented by Figure 1. To describe the scale of a sensor network, the longest path between two nodes is defined as the diameter $d_{g}$ of graph G.

### 2.3. Notations

Throughout this paper, the notations are kept standard. $R^{n_{x}}$ and $R^{m_{i}\times n_{x}}$ stand for the $n_{x}$-dimensional Euclidean space and set of all $m_{i}\times n_{x}$ matrices, respectively. The operator E(·) and ∥·∥ stands for the operation of expectation and norm, respectively. The superscript *T* stands for the transpose of a matrix. $x_{k|k-1}^{i}\left(\cdot \right)$ and $x_{k|k}^{i}\left(\cdot \right)$ stand for the prior and posterior estimates of state $x_{k}$ by sensor *i* at instant *k*, respectively.

## 3. Multi-Source Information Fusion Algorithm with Finite-Time Convergence

Building on the theoretical foundations introduced in Section 2, this section presents high-performance information fusion algorithm. For most multi-sensor information fusion scenarios, it is necessary to know the time or number of system iterations required to obtain stable fusion results. Therefore, to achieve fast and accurate acquisition of fusion results under a multi-source detection regime, this section presents a distributed Kalmanbased multi-source fusion algorithm with fast finite time convergence of fusion errors. Additionally, by introducing a matrix weighting algorithm, optimal estimation is achieved in the minimum variance sense.

To achieve dynamic correlation between fusion results and fusion process, inspired by references [4,33,34], the GIQ calculation method as adapted and introduced into the fusion algorithm proposed in this paper, to achieve dynamic information weighting during adjacent sensor information interaction. By calculating the bias of the local fusion results of the sensors at each moment, they are converted into dynamic information weights for information interaction $\omega_{ij}\left(k\right)$. Specifically:

(7) $${\mathrm{FB}}_{i}^{\chi }\left(k\right)=\left(x_{k|k}^{i}-x_{k}\right){\left(x_{k|k}^{i}-x_{k}\right)}^{T},$$

where ${\mathrm{FB}}_{i}^{\chi }\left(k\right)$ denotes the local fusion bias of sensor node *i* at instant *k*. χ denotes the state volume category of the state vector $x_{k}$, $x_{k}$ is the true state.

**Definition 1.**  *$\Delta_{d,ij}$ denote the relative deviation of the local fusion results for the i-th and j-th sensors, defined as:*

(8) $$\Delta_{d,ij}\left(k\right)=\sqrt{\frac{1}{2}{\left({\mathrm{FB}}_{i}^{\chi }\left(k\right)-{\mathrm{FB}}_{j}^{\chi }\left(k\right)\right)}^{T}I_{e}\left({\mathrm{FB}}_{j}^{\chi }\left(k\right)-{\mathrm{FB}}_{i}^{\chi }\left(k\right)\right)},$$

*where $I_{e}$ is the diagonal matrix:*

$$I_{e}={\left[\begin{array}{cccc} 1 & 0 & \cdots & 0\\ 0 & 1 & \cdots & 0\\ \vdots & \vdots & \ddots & 0\\ 0 & 0 & \cdots & 1 \end{array}\right]}_{n\times n}.$$

**Definition 2.**  *$S_{i}\left[{\mathrm{FB}}_{i}^{\chi }\left(k\right)\right]$ represent the support degree for the bias of the local fusion result of the i-th sensor, it has:*

(9) $$S_{i}\left[{\mathrm{FB}}_{i}^{\chi }\left(k\right)\right]=\sum_{j\in N_{i}}\left[1-\Delta_{d,ij}\left(k\right)\right].$$

**Definition 3.**  *From Definitions 1 and 2, let $G_{iq}\left[{\mathrm{FB}}_{i}^{\chi }\left(k\right)\right]$ denote the GIQ, which is defined by*

(10) $$G_{iq,i}\left[{\mathrm{FB}}_{i}^{\chi }\left(k\right)\right]=e^{\frac{S_{i}\left[{\mathrm{FB}}_{i}^{\chi }\left(k\right)\right]}{\sum_{i=1}^{n}S_{i}\left[{\mathrm{FB}}_{i}^{\chi }\left(k\right)\right]}}{∥{\mathrm{FB}}_{i}^{\chi }\left(k\right)∥}^{2},$$

*where ${∥{\mathrm{FB}}_{i}^{\chi }\left(k\right)∥}^{2}$ is the norm of vector ${\mathrm{FB}}_{i}^{\chi }\left(k\right)$.*

Next, it is necessary to normalize the calculation results of (10) and obtain the result as the dynamic weight for information interaction, that is:

(11) $$\omega_{ij}\left(k\right)=\frac{G_{iq,i}\left[{\mathrm{FB}}_{i}^{\chi }\left(k\right)\right]}{\sum_{i=1}^{n}G_{iq,i}\left[{\mathrm{FB}}_{i}^{\chi }\left(k\right)\right]}a_{ij}.$$

Regarding the design of Equations (7)–(11), a dynamic communication weight associated with the local fusion result has been developed, enhancing the correlation between the algorithm and the fusion process.

To achieve rapid convergence of fusion errors while simultaneously achieving the optimal estimate in the sense of minimum variance, the fusion algorithm with finite time convergence is summarized in Algorithm 1.
**Algorithm 1** Fusion Algorithm with Finite Time Convergence**Initialization:** **Step 1:** let k=1 and t=1, calculate $\psi_{k,t-1}^{i\to j}={\left(H_{k}^{i}\right)}^{T}{\left(R_{k}^{i}\right)}^{-1}y_{k}^{i}$, $\varphi_{k,t-1}^{i\to j}={\left(H_{k}^{i}\right)}^{T}{\left(R_{k}^{i}\right)}^{-1}H_{k}^{i}$. **Step 2:** let $x_{0|0}^{i}=\mu_{0}$, $P_{0|0}^{i}=P_{0}$, $\omega_{ij}\left(1\right)=1/\left|N_{i}\right|$. **Step 3:** Transmit $\psi_{1,0}^{i\to j}$ and $\varphi_{1,0}^{i\to j}$ to node *j*, where $j\in \{N_{i}\setminus \left\{i\right\}\}$.**Main loop:** **Step 4:** Calculate communication weights (12)$$\omega_{ij}\left(k\right)=f\left[{\mathrm{FB}}_{i}^{\chi }\left(k-1\right)\right].$$ **for** After iteration $t=1,2,\cdots ,d_{g}$, for each sensor *i*, and (i,j)∈E **do**
(13)$$\Theta_{i}\left(t\right)={\left(H_{k}^{i}\right)}^{T}{\left(R_{k}^{i}\right)}^{-1}y_{k}^{i}+\sum_{j\in N_{i}}\omega_{ij}\left(k\right)\psi_{k,t-1}^{j\to i},$$
(14)$$\Omega_{i}\left(t\right)={\left(H_{k}^{i}\right)}^{T}{\left(R_{k}^{i}\right)}^{-1}H_{k}^{i}+\sum_{j\in N_{i}}\omega_{ij}\left(k\right)\varphi_{k,t-1}^{j\to i}.$$      For each sensor $j\in N_{i}$,
(15)$$\begin{array}{c} \psi_{k,t}^{j\to i}=\Theta_{i}\left(t\right)-\psi_{k,t-1}^{i\to j},\\ \varphi_{k,t}^{j\to i}=\Omega_{i}\left(t\right)-\varphi_{k,t-1}^{i\to j}. \end{array}$$ **end for** **Step 5:** Calculate $x_{k|k}^{i}$ and $P_{k|k}^{i}$ by (16)$$x_{k|k-1}^{i}=\Phi_{k-1}x_{k-1|k-1}^{i},$$
(17)$$P_{k|k-1}^{i}=\Phi_{k-1}P_{k-1|k-1}^{i}\Phi_{k-1}^{T}+Q_{k-1},$$
(18)$${\left[P_{k|k}^{i}\right]}^{-1}={\left[P_{k|k-1}^{i}\right]}^{-1}+\Omega_{i}\left(d_{g}\right),$$
(19)$$\begin{array}{c} x_{k|k}^{i}=P_{k|k}^{i}\left[{\left(P_{k|k-1}^{i}\right)}^{-1}x_{k|k-1}^{i}+\Theta_{i}\left(d_{g}\right)\right]. \end{array}$$ **Step 6:** Optimal weighted fusion **for**
k≥1
**do**  (20)$x_{k|k}^{f}=\Gamma_{1}x_{k|k}^{1}+\Gamma_{2}x_{k|k}^{2}+\cdots +\Gamma_{n}x_{k|k}^{n}.$ **end for**

$x_{k|k}^{i}$ as the local estimation result of sensor *i* on the system state variable *x* at instant *k*, $P_{k|k}^{i}$ is the fusion error covariance of estimator *i* at instant *k*, $x_{k|k}^{f}$ is the optimal fusion result with respect to $x_{k}$ in the minimum variance sense. f(·) is the GIQ calculation operator, which can be referred to as (7)–(11).

**Remark 1.**  *To facilitate the understanding of the execution process and design principles of Algorithm 1, the following explanations were provided:regarding the initialization steps 1 to 3. The main tasks were to compute the information propagation factors $\psi_{k,t-1}^{i\to j}$, $\varphi_{k,t-1}^{i\to j}$ and assign initial values to the fusion algorithm. In step 1 of the main loop, dynamic weight coefficients $\omega_{ij}\left(k\right)$ were computed using (7)–(11). These coefficients were used to assign weights for the subsequent information exchange among sensor nodes. For (13) and (14), they respectively achieved the aggregation of sensor measurement information and sensor status information towards a single sensor node i. During the finite number of iterations (the diameter of graph $d_{g}$), each node possesses global information. Additionally, considering the issue of information quality during the information transmission process, the communication weight $\omega_{ij}\left(k\right)$ was incorporated. (16)–(19) represent the classical local Kalman filtering method. (16) and (17) were the state prediction equations, and (18) and (19) were the state update equations. Finally, the local filtering results obtained from Equation (19) are combined using matrix weighted fusion, as described in Equation (20).*

**Remark 2.**  *In Algorithm 1, the inputs include the target information $y_{k}^{i}$ measured by sensors, as well as parameters $\psi_{k,t-1}^{i\to j}$ and $\varphi_{k,t-1}^{i\to j}$ transmitted from neighboring sensors. The constraints involve the graph diameter $d_{g}$ and the number of nodes in the sensor network n. The feedback includes the bias ${\mathrm{FB}}_{i}^{\chi }\left(k\right)$ of the local fusion result from the sensors, and the output is the optimal fusion result $x_{k|k}^{f}$.*

**Theorem 1.**  *Considering the information transmission network system between sensors in (1) and (2), assuming that the communication topology diagram G is an undirected tree diagram with a diameter of $d_{g}$, optimal fusion results $x_{k|k}^{f}$ in terms of minimum variance can be obtained through Algorithm 1.*

**Proof.**  Given the system Model (1) and the measurement Model (2), the local KF can be expresses as: [24]

Update:

(21) $$x_{k|k}^{i}=x_{k|k-1}^{i}+K_{k}^{i}\left[y_{k}^{i}-H_{k}^{i}x_{k|k-1}^{i}\right],$$

(22) $$P_{k|k}^{i}=P_{k|k-1}^{i}K_{k}^{i}H_{k}^{i}P_{k|k-1}^{i},$$

(23) $$K_{k}^{i}=P_{k|k-1}^{i}{\left(H_{k}^{i}\right)}^{T}{\left[H_{k}^{i}P_{k|k-1}^{i}{\left(H_{k}^{i}\right)}^{T}+R_{k}^{i}\right]}^{-1}.$$

Prediction:

(24) $$x_{k+1|k}^{i}\left(s\right)=\Phi_{k}x_{k|k}^{i},$$

(25) $${\hat{P}}_{k|k+1}^{i}=\Phi_{k}P_{k|k}^{i}\Phi_{k}^{T}+Q_{k}.$$

where $x_{k+1|k}^{i}$ and $P_{k+1|k}^{i}$ are one-step priori state estimator and estimate error covariance, respectively.

Perform matrix inverse operations on (21) and (22) respectively, it is obtained that:

(26) $$x_{k|k}^{i}=P_{k|k}^{i}\left({\left(P_{k|k-1}^{i}\right)}^{-1}x_{k|k-1}^{i}+\sum_{i\in N_{i}}{\left(H_{k}^{i}\right)}^{T}{\left(R_{k}^{i}\right)}^{-1}y_{k}^{i}\right),$$

(27) $${\left(P_{k|k}^{i}\right)}^{-1}={\left(P_{k|k-1}^{i}\right)}^{-1}+\sum_{i\in N_{i}}{\left(H_{k}^{i}\right)}^{T}{\left(R_{k}^{i}\right)}^{-1}H_{k}^{i}.$$

To dynamically correlate the fusion results with the fusion process as mentioned earlier in this section, (26) can be rewritten as:

(28) $$x_{k|k}^{i}=P_{k|k}^{i}\left[{\left(P_{k|k-1}^{i}\right)}^{-1}x_{k|k-1}^{i}+\Theta_{i}\left(d_{g}\right)\right],$$

where $\Theta_{i}\left(d_{g}\right)=\sum_{j\in N_{i}}\psi_{k,d_{g}-1}^{j\to i}$, it can be obtained by iterating $\psi_{k,0}^{j\to i}$ for $d_{g}$ times. And $\psi_{k,0}^{j\to i}={\left(H_{k}^{i}\right)}^{T}{\left(R_{k}^{i}\right)}^{-1}y_{k}^{i}$. Obviously, $\omega_{ii}\left(k\right)=1$, one has that

(29) $$\Theta_{i}\left(t\right)={\left(H_{k}^{i}\right)}^{T}{\left(R_{k}^{i}\right)}^{-1}y_{k}^{i}+\sum_{j\in N_{i}}\omega_{ij}\left(k\right)\psi_{k,t-1}^{j\to i}.$$

Similarly, with the above analysis for (27), it can be obtained that:

(30) $${\left(P_{k|k}^{i}\right)}^{-1}={\left(P_{k|k-1}^{i}\right)}^{-1}+\Omega_{i}\left(d_{g}\right),$$

where $\Omega_{i}\left(t\right)={\left(H_{k}^{i}\right)}^{T}{\left(R_{k}^{i}\right)}^{-1}H_{k}^{i}+\sum_{j\in N_{i}}\omega_{ij}\left(k\right)\varphi_{k,t-1}^{j\to i}$ and $\varphi_{k,0}^{j\to i}={\left(H_{k}^{i}\right)}^{T}{\left(R_{k}^{i}\right)}^{-1}H_{k}^{i}$.

(28) and (30) obtain the locally fused state estimate and covariance, which need to be further fused. To achieve the optimal estimation in terms of minimum variance, matrix weight fusion coefficients are introduced.

For (20), assuming the unbiasedness of the fusion process, it has $E\left(x_{k|k}^{i}\right)=E\left(x_{k}\right)$. Taking the expected value of both sides of (20), we can obtain:

(31) $$I_{n}=\Gamma_{1}+\Gamma_{2}+,\ldots ,+\Gamma_{n}.$$

Let ${\hat{x}}_{k}=\sum_{i=1}^{n}\Gamma_{i}\left(x_{k}-x_{k|k}^{i}\right)$, from [10], the cross-covariance of local filtering between sensor *i* and *j* can be expressed as $P_{k}^{ij}=\left(I_{n}-K_{k}^{i}H_{k}^{i}\right)\left(\Phi_{k-1}P_{k-1}^{ij}\Phi_{k-1}^{T}+Q_{k-1}\right)\left(I_{n}-K_{k}^{j}H_{k}^{i}\right)$. To the objective of achieving optimal estimation in terms of minimum variance set in this paper, the performance indicator for the estimation algorithm is:

(32) $$J_{k}=\sum_{i,j=1}^{l}\mathrm{tr}(\Gamma_{i}P_{k}^{ij}\Gamma_{j}^{T}).$$

The optimization objective is set to minimize (32) under the constraint of (31), which in turn is solved to obtain:

(33) $$\left(\begin{array}{c} \Gamma \\ U \end{array}\right)={\left(\begin{array}{cc} \Sigma & e\\ e^{T} & 0 \end{array}\right)}^{-1}\left(\begin{array}{c} 0\\ I_{n} \end{array}\right)=\left(\begin{array}{c} \Sigma^{-1}e{\left(e^{T}\Sigma^{-1}e\right)}^{-1}\\ -{\left(e^{T}\Sigma^{-1}e\right)}^{-1} \end{array}\right).$$

Therefore, from the above equation, the optimal matrix weight $\Gamma =\Sigma^{-1}e{\left(e^{T}\Sigma^{-1}e\right)}^{-1}$ can be obtained, and the corresponding minimum covariance is $P_{k|k}^{f}={\left(e^{T}\Sigma^{-1}e\right)}^{-1}$, that is: $\mathrm{tr}\left(P_{k|k}^{f}\right)\leq \mathrm{tr}\left(P_{k}^{ii}\right)$, where $\Sigma ={\left(P_{k}^{ij}\right)}_{n_{x}\times n}$, i,j=1,2,⋯,n is a symmetric positive definite matrix. Detailed proof process can be found in [10].  □

**Remark 3.**  *Based on the above analysis, it is evident that the computation cost of matrix weight Γ is related to the number of sensors and the measured states. When the number of sensors is large, the data fusion process with weighted data can introduce computational burden and affect the real-time performance of the fusion effect. To achieve the optimal fusion in terms of minimum variance and reduce computational cost, an improvement can be made to Σ such that $\Sigma ={\left({\tilde{P}}_{k}^{ij}\right)}_{n_{x}\times n}$,i,j=1,2,⋯,n, where ${\tilde{P}}_{k}^{ij}$ are the diagonal matrix constructed from the diagonal elements of the covariance matrix $P_{k}^{ij}$.*

Theorem 1 provides the first characteristic of the proposed algorithm in this paper, it can achieve optimal estimation in terms of minimum variance. The following theorem will provide the second characteristic of the algorithm: finite-time convergence of fusion error, which means that after a finite number of algorithm iterations, the fusion error converges.

**Theorem 2.**  *Considering the information transmission network system between sensors in (1) and (2), assuming that the communication topology diagram G is an undirected tree diagram with a diameter of $d_{g}$, Algorithm 1 can achieve convergence of sensor fusion error after $d_{g}$ iterations.*

**Proof.**  Since the communication topology graph G of the sensor network is an undirected tree graph, it can be decomposed into two subtree graphs $G_{i}$ and $G_{j}$ connected by sensor nodes (i,j). Sensor node *i* and *j* are the root nodes of subtree graphs $G_{i}$ and $G_{j}$, respectively.

To achieve convergence of fusion error in finite time when using fusion Algorithm 1 for target state estimation in sensor networks, the following steps need to be taken:

Step 1: By definition, in the subgraph $G_{i}$, the level of a sensor node is defined as the number of hops from the sensor node to the root node *i*. Obviously, node *i* is called the layer-0 node; if a node is only one hop away from the root node *i*, then these nodes are called level-1 nodes, and so on.

Step 2: When t=1, to obtain an estimate of the target state $x_{k}$, according to (13) and (14), and the initial step of Algorithm 1, only the information from the layer-0 nodes in graph $G_{i}$ is used. The sensor detection information from other sensor nodes in subgraph $G_{i}$ has not been utilized yet.

Step 3: According to the analysis process in step 2, this means that after $d_{g}$ iterations, using the sensor nodes in subgraph $G_{i}$ from layer-0 to layer-$d_{g}$, the sensing information can be transmitted to $\psi_{k,d_{g}}^{i\to j}$ and $\varphi_{k,d_{g}}^{i\to j}$, allowing them to have global information and obtain accurate target state.

Step 4: Similarly, in subgraph $G_{j}$, due to the adjacency between sensor nodes *i* and *j*, through $d_{g}-1$ iterations and utilizing the sensing information from sensor nodes in layer-0 to layer-$d_{g}-1$, $\psi_{k,d_{g}}^{j\to i}$ and $\varphi_{k,d_{g}}^{j\to i}$ can obtain the information required for target state estimation.

Step 5: By utilizing the information from subgraphs $G_{i}$ and $G_{j}$, it is obtained that

(34) $$\Omega_{i}\left(d_{g}\right)=\varphi_{k,d_{g}}^{i\to j}+\varphi_{k,d_{g}-1}^{j\to i},$$

(35) $$\Theta_{i}\left(d_{g}\right)=\psi_{k,d_{g}}^{i\to j}+\psi_{k,d_{g}-1}^{j\to i}.$$

Finally, by substituting the obtained $\Omega_{i}\left(d_{g}\right)$, $\Theta_{i}\left(d_{g}\right)$ into (18) and (19) respectively, we can obtain the posterior estimate of the target state and the estimation error covariance.   □

The proof of Theorem 2 illustrates the main idea behind the finite-time convergence of fusion errors in Algorithm 1. By iteratively transmitting the information propagation factors $\psi_{k,t-1}^{i\to j}$ and $\varphi_{k,t-1}^{i\to j}$ of each sensor in the network, the algorithm achieves the aggregation of individual node information, leading to the global fusion information.

In summary, with the support of Algorithm 1, a sensor network can utilize a limited number of data iterations to enable each sensor node to obtain target information detected by other sensors in the network. This allows the filtering results to have global coverage, resulting in accurate target state estimation. The number of iterations required is equal to the diameter ($d_{g}$) of the graph G, which is also the minimum number of iterations needed for the estimation error to converge in finite time.

**Remark 4.**  *In real-world scenarios of sensor networks, various interferences exist that can affect the stability of information transmission in the network. In order to reduce communication burden, the proposed fusion algorithm should be capable of being applied to directed graphs. According to the design concept of Algorithm 1, it is required that when the communication topology graph is a directed graph, convergence of fusion errors within a finite time can be achieved if and only if there exists a directed path from each sensor node to every other sensor node. This condition indicates that the directed graph is strongly connected, and Algorithm 1 can then accomplish the convergence of fusion errors in finite time.*

## 4. Numerical Examples and Experiments

### 4.1. The Simulation of Fusion Algorithm with Finite Time Convergence

**Figure 2:**
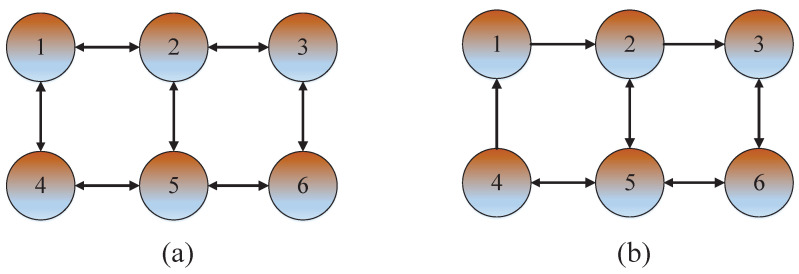
Structure of the sensor network. (**a**) undirected graph, (**b**) directed graph.

In this subsection, performance verification will be conducted on Algorithm 1 for sensor network communication topologies with undirected and directed graphs. Considering a detection and tracking system with six sensors, where two sensors are used for target displacement measurement, two sensors for velocity measurement, and the remaining two sensors for acceleration measurement. The discrete-time system can be represented as follows:

(36) $$x_{k+1}=\left[\begin{array}{ccc} 1 & T & T^{2}/2\\ 0 & 1 & T\\ 0 & 0 & 1 \end{array}\right]\;\;x_{k}+w_{k},$$

where, T=0.01 s is the sampling period, and the target state vector is $x_{k}={\left[s_{k},{\dot{s}}_{k},{\ddot{s}}_{k}\right]}^{T}$, $s_{k}$, ${\dot{s}}_{k}$ and ${\ddot{s}}_{k}$ are the target position, velocity and acceleration, respectively. The parameters of the observation equation are $H^{1}=\left[1,0,0\right]$, $H^{2}=\left[0,1,0\right]$, $H^{3}=\left[0,0,1\right]$, $H^{4}=\left[1,0,0\right]$, $H^{5}=\left[0,1,0\right]$, $H^{6}=\left[0,0,1\right]$, Q=0.8 and $R^{i}=\mathrm{diag}\left[0.9,1.1,1.2,1.0,0.7,1.0\right]$ at any given time. The initial state of target are $\mu_{0}=\left[0,\ldots ,0\right]$ and $P_{0}=I_{6}$, where $I_{6}$ is a 6-dimensional diagonal matrix with all elements being 1. The network connectivity among the sensors is shown in Figure 2. And different numbers represent different sensor nodes.

**Figure 3:**
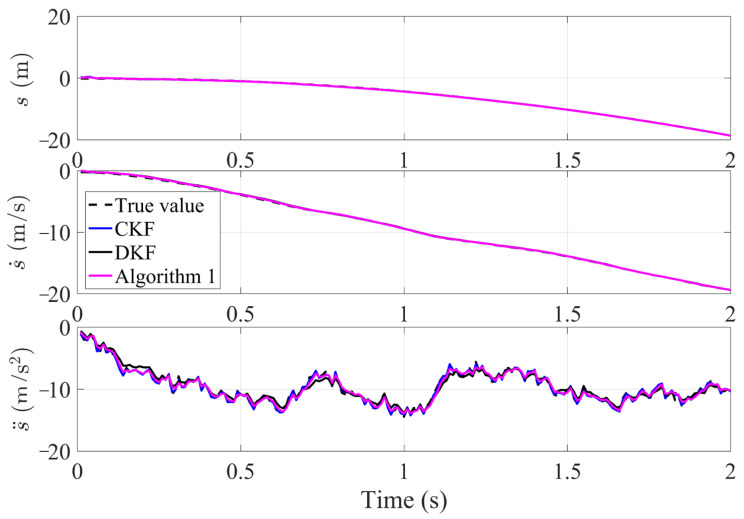
Tracking effect of Algorithm 1 under undirected graph.

As shown in Figure 3, the tracking effect of Algorithm 1, the CKF and DKF algorithm in this paper for the same target in the undirected graph (a). From top to bottom, the tracking effects of target position, velocity and acceleration are shown respectively. It can be seen that the tracking results of the filter algorithm proposed in this paper are very close to those of the CKF algorithm, which has the ability to track targets with high accuracy. Careful observation reveals that the tracking accuracy of the algorithm proposed in this paper is superior to that of the classical DKF algorithm, particularly in terms of stability. To make a clearer comparison the tracking errors of algorithms, the mean squared error (MSE) is used to characterize them. It is calculated as: $\mathrm{MSE}=\frac{1}{n}\sum_{i=1}^{n}{\left(x_{k|k}^{f}-x_{k}\right)}^{2}$.

**Figure 4:**
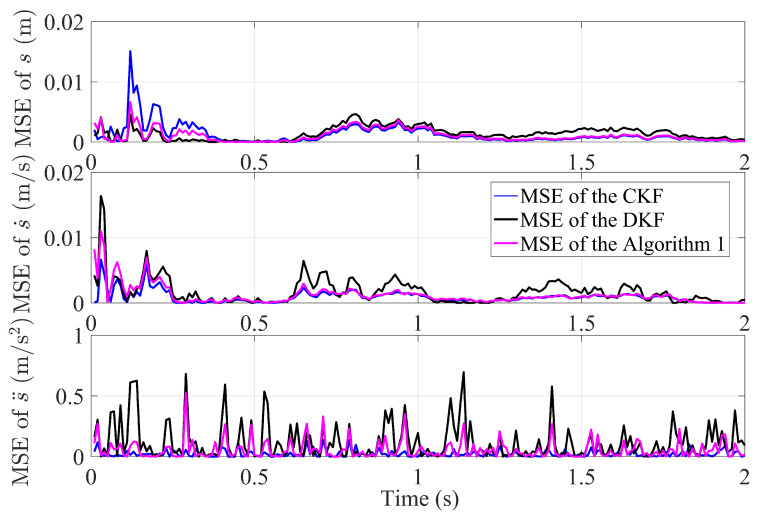
The comparative MSE performance between Algorithm 1, CKF and DKF algorithm in an undirected graph.

Based on the calculation results of MSE, Figure 4 shows the MSE comparison between the proposed Algorithm 1, DKF and CKF algorithm in this paper. The MSE of all algorithms is very small, which means they all have excellent tracking performance. By careful observation, after a finite number of iterations over time, the information from each sensor is transmitted to the other sensors through the sensor network, the MSE of Algorithm 1 gradually stabilizes. It is evident that the MSE of the proposed Algorithm 1 is smaller and more stable than DKF. This is attributed to the introduction of the finite-time control strategy and the optimal matrix weighting technique.

**Figure 5:**
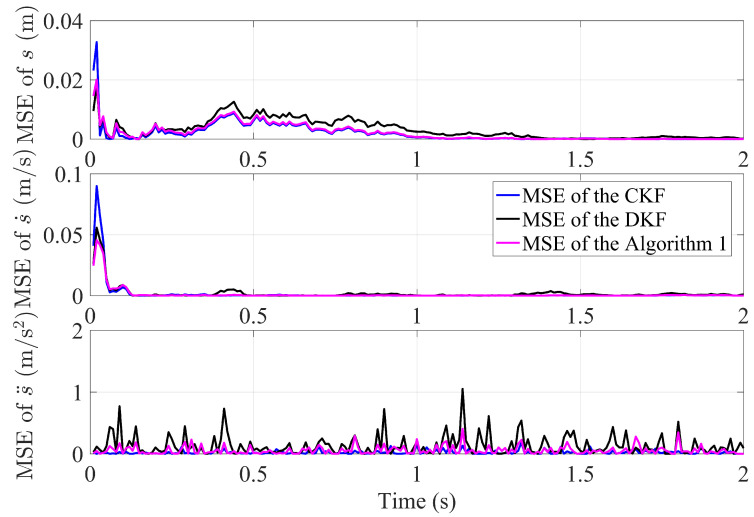
The comparative MSE performance between Algorithm 1, CKF and DKF algorithm in a directed graph.

In addition, Figure 5 compares the MSE of Algorithm 1, DKF and CKF algorithm for tracking the same target when the sensor network is the directed graph (b). It can be observed that when the sensor network is a directed graph, the MSE of the estimated position, velocity, and acceleration of the target increases due to the influence of harsh communication conditions. However, the increase in MSE is still small, indicating that the algorithm proposed in this paper can maintain tracking accuracy.

In summary, through the aforementioned target tracking simulation verification, it can be observed that the Algorithm 1 proposed in this paper, achieves tracking performance similar to the CKF and outperforms the classical DKF. Even in directed graphs with poor communication, it can maintain good filtering results.

### 4.2. Experiments of Fusion Algorithm with Finite Time Convergence

#### 4.2.1. Static Target Testing

**Figure 6:**
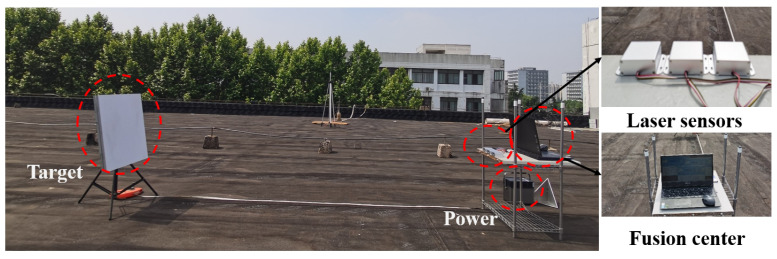
Multi-source detection of static targets.

In this subsection, sensor-measured data will be utilized for Algorithm 1 feasibility verification. The testing scenario and the sensors used are shown in Figure 6. The measurement range of the laser ranging sensor (GJD-01) is 0.045 to 100 m, with a measurement error of 0.05 m.

To avoid measurement bias caused by random errors in sensors during the testing process, multiple laser ranging sensors are used to measure the same distance multiple times. The outliers in the data obtained from the same sensor are removed, and the average value is calculated as the final measured distance for that sensor at the current distance. Similarly, relative distance measurements between multiple groups of sensors and stationary targets are conducted at different distances.

By calculating the average of the distance measurements from each sensor in Table 2 and inputting them into Algorithm 1, i.e., $y_{k}^{i}$, where i=1,2,3 corresponds to the data in Table 2, the sensor measurement variances are $R_{1}=R_{2}=R_{3}=10^{-4}$. The estimated covariance *Q* is set to 0.5. It can be observed that the sensor measurements are more accurate than the estimates, so the final fusion result will be biased towards the actual sensor measurements. The fusion result can be seen in the last row of Table 2, indicating a close fusion accuracy to the true distance value, consistent with the simulation analysis in Section 4.1. Moreover, the fusion result demonstrates an improvement in measurement accuracy compared to the individual sensor measurements. Therefore, the multi-source fusion algorithm designed in this paper possesses high-precision measurement fusion capability.

#### 4.2.2. Mobile Target Testing

**Figure 7:**
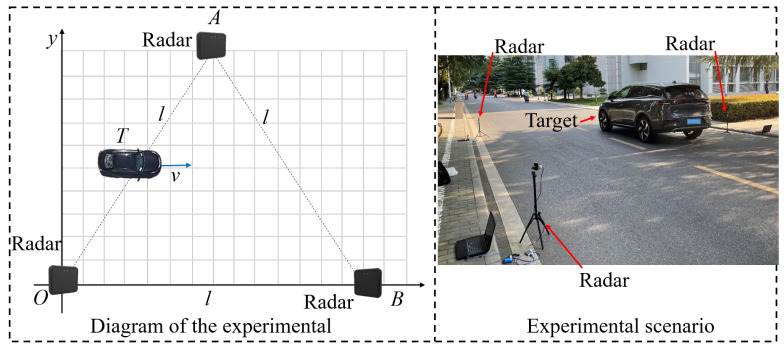
Experimental setup.

To verify the fusion performance of Algorithm 1 in observing moving targets, an experimental setup was arranged as depicted in Figure 7. Using the moving target plane as the coordinate system, three 24 G millimeter-wave radar sensors (Nanoradar SP25 millimeter-wave radar) were situated at coordinates (0, 0) m, (5, 0) m, and ($\frac{5}{2}$, $\frac{5}{2}\sqrt{3}$) m, respectively. The target originated from the position (0, $\frac{7}{2}$) m and moved at a nearly uniform velocity parallel to the positive *x*-axis. Each sensor primarily measured the target’s relative distance and relative bearing, which were then transformed to the origin (0, 0) at the same location using the sine theorem.

**Figure 8:**
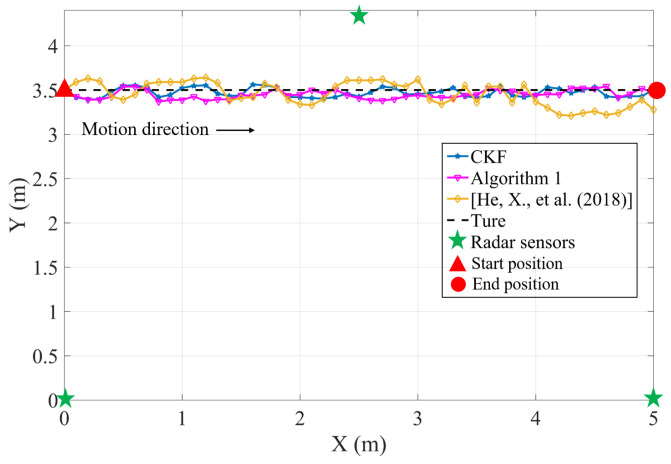
Experimental result compared to [35].

**Figure 9:**
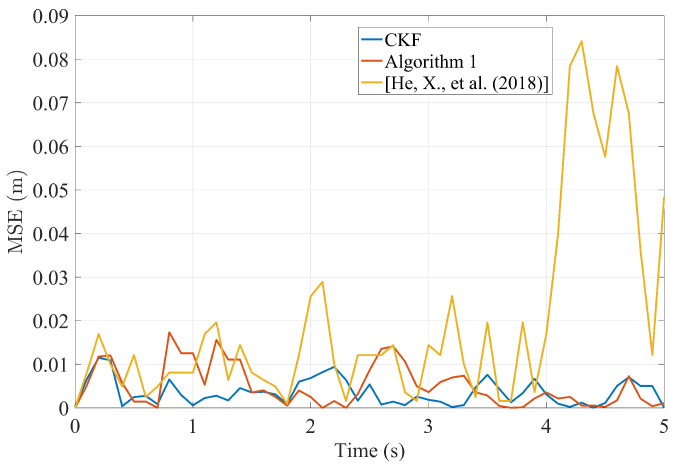
Position MSE with multi-sensor fusion compared to [35].

The experiment consists of 51 samples with sampling interval *T* = 0.1 s. As shown in Figure 8 and Figure 9, the experimental results and the MSE for relative position tracking are presented. A comparison is made among the tracking performances of the CKF, Algorithm 1, and the approach proposed in [35].

As can be seen from Figure 8, Algorithm 1 designed for a distributed sensor network environment in this study achieves accurate tracking of moving targets. In terms of tracking accuracy, it closely approaches the performance of the CKF and outperforms the algorithms proposed in [35]. Particularly noteworthy is the outstanding stability of the filtering results exhibited by the algorithm proposed in this paper.

Figure 9 presented the results for MSE of relative positions. Consistent with the previous analysis, the Algorithm 1 proposed in this paper demonstrates fusion results comparable to CKF. Moreover, the maximum MSE didn’t exceed 0.02 m, in contrast to the maximum 0.09 m MSE reported in [35]. Thus, the optimally designed distributed finite-time fusion method presented in this paper exhibits exceptional performance.

## 5. Conclusions

To meet the precise sensing requirements of sensor networks, a novel optimal distributed finite-time fusion filtering method was developed in this study. Firstly, by combining finite-time control techniques and graph theory, a multi-source information fusion method was designed to achieve rapid convergence of fusion error within a limited number of iterations. Based on this, matrix weights were embedded to achieve the optimal estimation of fusion results in terms of minimum variance. Next, the filtering method designed in this study focuses on the fusion process. It utilizes the local filtering results to obtain the bias and combines it with the GIQ calculation method to generate dynamic communication weights embedded in the fusion algorithm. Finally, numerical simulations, static distance detection fusion, and dynamic target tracking experiments were conducted to validate the effectiveness and feasibility of the proposed variance. In future work, the impact of information transmission delay on multi-source information fusion will be considered.

## Figures and Tables

**Table 1:** Performance comparison of main methods.

Methods	The Main Pros	The Main Cons
CKF [10,24]	High fusion accuracy	Poor robustness
DKF [25]	Strong trade-off between robustness and plasticity	Fusion accuracy is lower than CKF
Average-DKF [11,12]	Convergence of fusion result error	The number of iterations for convergence is unknown
finite-time DKF [15,18]	The number of iterations for convergence is known	The information interaction process is not dynamically correlated with the fusion process
Weight-DKF [19,20]	Dynamic fusion weights	Weight settings only focus on the final fusion process

**Table 2:** Relative distance measurement data recording and fusion results for stationary target.

	Sensors	Distance/m				
		1	2	5	10	20
Sensor measurement data/m	Sensor 1	1.014	1.996	4.964	9.983	19.970
		0.988	2.001	5.020	10.011	19.964
		1.014	1.988	4.974	9.984	20.172
		1.086	2.082	4.975	9.981	19.968
		0.990	1.990	5.004	10.029	20.066
	Sensor 2	0.988	2.025	4.979	10.011	19.977
		1.069	2.087	5.069	9.959	20.043
		1.087	1.990	4.970	9.965	19.979
		0.973	1.979	4.977	10.091	20.082
		1.070	2.041	5.037	9.979	20.021
	Sensor 3	0.981	1.992	5.011	9.943	19.941
		0.985	2.012	4.987	10.012	20.058
		1.047	1.982	5.006	9.938	20.029
		0.985	1.987	4.974	10.042	19.927
		1.082	2.010	4.981	9.941	20.019
Fusion result/m		1.006	2.008	4.990	9.999	20.007

## Data Availability

Not applicable.
